# *Externalising *and *emotional *categories, diagnostic groups and clinical profiles

**DOI:** 10.1186/1752-4458-4-20

**Published:** 2010-07-15

**Authors:** Graham W Mellsop, Alison Bower, Sandra L Baxendine

**Affiliations:** 1 Waikato Clinical School, University of Auckland, New Zealand; 2 Te Pou, Kakariki House 293 Grey Street, P O Box 219, Hamilton, New Zealand

## Abstract

**Background:**

It has been proposed that gains would be made in the validity of the psychiatric classification system if many of the present 'neurotic' or personality disorders were subsumed into two over-arching groups, externalising and emotional disorders. If diagnostic sub-categories from the first digit coding structures within ICD-10 do, in fact, share clinical phenomenology that align with the major externalising/emotional distinction, this further supports the proposal and contributes to face validity. The aim of the study was to examine the distribution of particular psychopathology within and between two proposed over-arching groupings - externalising and emotional disorders - in a clinical sample.

**Method:**

The distributions of HoNOS derived information in relation to the proposed clusters of emotional disorders and extrinsic disorders are examined.

**Results:**

Statistically significant differences in profiles between the emotional and the externalising groupings are consistent with the proposed classification development. The HoNOS (Health of Nation Outcome Scale) measures of self harm, depression, aggression, occupational/leisure problems and drug and alcohol consumption are the five most significant discriminators between the two groups.

**Discussion:**

The details of the profile differences within the two over arching groups suggest that further examination is required. Useful work could include examination in credibly large and unselected patient populations of the factor structure demonstrated in non patient samples. Prospective comprehensive trials of the contributions the proposed classification could make to clinical decision making would also help illuminate this area.

## Background

In this period of development of new versions of the International Classification of Diseases (replacing 10^th ^Edition) and of the Diagnostic and Statistical Manual of the American Psychiatric Association (replacing DSM-IV TR) there is much debate about the utility and validity of their present overall structures [1-10]. Some have proposed that validity gains can be achieved by subsuming many of the present affective, neurotic, personality and substance abuse categories into two over-arching groups, externalising and emotional disorders [11-15]. While earlier publications had spoken of "internalising" disorders, the name for this group has evolved to "emotional". "Internalising" suggested too strongly an aetiological implication, which might be particularly misunderstood by the broader, non-clinician, community. "Emotional" has a credible history as a classificatory term.

The five proposed contributory disorders for the externalising group are alcohol related disorders, drug disorders, anti-social personality disorder, borderline personality disorder and ADHD. The proposed constituent disorders for the emotional group are anxious avoidant/dependent personality, major depression, dysthymia, general anxiety disorder, agoraphobia, social phobia, specific phobia, panic disorder, PTSD, obsessive compulsive disorder.

The majority of published research supporting the factorial integrity of such a distinction comes from studies of non-clinical populations, where either structured interview systems have been applied to a community sample, or more specific psychological tests have been used in random community samples. If diagnostic sub-categories from the first digit coding structures within ICD-10 do have clinical phenomenology commonalities that align with an externalising/emotional distinction it would further support the utility of this model and contribute to the 'face validity' of the construct.

The HoNOS is widely used to examine the outcomes of patient care in Mental Health Services. It does that by allowing repeated, standardised assessment of a range of mental status phenomena and some functionality items [16].

The 12 clinical information items covered by the HoNOS are:-

1. Overactive, aggressive, disruptive or agitated (Agr)

2. Non-accidental self injury (Self Harm)

3. Problem drinking or drug taking (AoD)

4. Cognitive problems (Cog)

5. Physical illness or disability (Phy)

6. Problems with hallucinations and/or delusions (Del/Hal)

7. Problems with depressed mood (Dep)

8. Other mental and behavioural problems (Other)

9. Relationship problems (Relat)

10. Problems with activities of daily living (ADL)

11. Problems with living conditions (Living)

12. Problems with occupation/leisure (Occup)

In New Zealand the routine collection of HoNOS data on public mental health service users is a policy expectation [17]. There have been a number of projects in New Zealand utilising HoNOS items as standardised assessment recordings to explore the distribution of patients presenting with particular phenomena in relation to other variables [e.g., [18-20]].

The paper seeks to answer a simple but important question: In a clinical population are disorders that can be grouped into the two categories (externalising/emotional) associated with different HoNOS profiles?

## Methods

The data were collected from five health service districts participating in a Test Project managed by Te Pou Information [21] in New Zealand. The HoNOS assessments for service users (18-64 years) were collected over a 2 year period (1 January 2007- 2009). The highest HoNOS total item score (within the year of diagnosis) and the most recent diagnosis, provided the data for analysis.

In total 4,238 patients had diagnoses representive of the proposed externalising and emotional groupings where the HoNOS record showed at least one "clinically significant" item. After assessments by Drug & Alcohol teams are excluded the number reduced to 3,212 (2,668 community assessments, 544 inpatient assessments).

Statistical analyses included the Mann-Whitney U test comparing rankings between the two groups of externalising and emotional disorder diagnoses. The Kruskal-Wallis was then used to compare the ranks within each of the emotional and externalising groups (methods appropriate for non-parametric data such as individual HoNOS scores).

## Results

Table 1 shows mean scores on each of the 12 HoNOS items separately for the externalising and emotional groups. The difference between mean scores is statistically significant for each HoNOS item except item 5 (Physical illness or disability problems). The five most notable significant differences are in higher measures of depression for the emotional group and drug and alcohol consumption, self harm, occupational or leisure difficulties and aggression in the externalising disorder group.

**Table 1:** HoNOS item means and standard deviations for the two proposed diagnostic clusters

HoNOS Items	Externalising Disorders Mean (SD)	Emotional Disorders Mean (SD)	P value
Agr	1.2 (1.2)	0.7 (0.9)	**p < 0.001**
Self Harm	1.2 (1.4)	0.7 (1.2)	**p < 0.001**
AoD	2.1 (1.5)	0.6 (1.0)	**p < 0.001**
Cog	0.7 (1.0)	0.4 (0.8)	**p < 0.001**
Phy	0.8 (1.1)	0.7 (1.1)	0.087
Del/Hal	0.6 (1.1)	0.2 (0.7)	**p < 0.001**
Dep	1.7 (1.2)	2.2 (1.1)	**p < 0.001**
Other	1.8 (1.4)	2.2 (1.2)	**p < 0.001**
Relat	1.9 (1.1)	1.5 (1.1)	**p < 0.001**
ADL	1.1 (1.2)	0.8 (1.0)	**p < 0.001**
Living	0.8 (1.2)	0.4 (0.8)	**p < 0.001**
Occup	1.1 (1.3)	0.6 (1.0)	**p < 0.001**

Total	433	2,779	

Mean HoNOS item scores for each diagnostic category contributing to the externalising disorder group are displayed in Table 2 and for the emotional disorder group in Table 3. Of particular interest are the following results:

**Table 2:** HoNOS item means and standard deviations for ADHD, Adult Antisocial, Alcohol Disorder, Borderline Personality Disorder and Drug Disorder groups

	ADHD	Adult Antisocial Disorder	Alcohol Disorder	Borderline Personality Disorder	Drug Disorder	
HoNOS Items	Mean (SD)	Mean (SD)	Mean (SD)	Mean (SD)	Mean (SD)	P value
Agr	1.2 (0.8)	1.9 (1.1)	1.1 (1.2)	1.0 (1.0)	1.4 (1.3)	**0.019**
Self Harm	0.2 (0.8)	1.0 (1.3)	1.2 (1.5)	1.8 (1.4)	0.7 (1.2)	**p < 0.001**
AoD	0.4 (0.9)	2.0 (1.3)	2.8 (1.3)	0.9 (1.2)	2.6 (1.2)	**p < 0.001**
Cog	1.4 (1.0)	0.9 (1.3)	0.9 (1.1)	0.4 (0.7)	0.8 (1.0)	**p < 0.001**
Phy	0.4 (0.7)	1.1 (1.4)	0.8 (1.1)	1.1 (1.2)	0.6 (1.0)	**0.014**
Del/Hal	0 (0)	0.6 (1.0)	0.4 (0.9)	0.3 (0.8)	1.0 (1.3)	**p < 0.001**
Dep	0.9 (1.0)	1.4 (1.2)	1.9 (1.2)	2.1 (1.0)	1.4 (1.2)	**p < 0.001**
Other	2.3 (0.9)	2.0 (1.3)	1.7 (1.5)	2.0 (1.2)	1.8 (1.4)	0.254
Relat.	1.2 (0.9)	2.3 (1.1)	1.9 (1.3)	2.0 (1.0)	1.8 (1.2)	0.056
ADL	0.9 (1.0)	0.9 (1.2)	1.3 (1.4)	0.9 (1.1)	1.1 (1.2)	0.410
Living	0.2 (0.4)	0.8 (0.9)	0.9 (1.3)	0.5 (0.9)	1.0 (1.3)	**0.002**
Occup	0.8 (1.2)	0.7 (1.0)	1.3 (1.4)	0.9 (1.1)	1.1 (1.3)	0.143

Total	13	18	138	137	127	

**Table 3:** HoNOS item means and standard deviations for the Contributory Emotional Disorders

	Agora-phobia	Anxious Avoidant Dependant	Dysthymia	General Anxiety Disorder	Major Depression	OCD	Panic Disorder	Post-Traumatic Stress Disorder	Social Phobia	Specific Phobia	P value
HoNOS Items	Mean (SD)	Mean (SD)	Mean (SD)	Mean (SD)	Mean (SD)	Mean (SD)	Mean (SD)	Mean (SD)	Mean (SD)	Mean (SD)	
Agr	0.6 (0.8)	1.3 (1.3)	0.8 (1.0)	0.6 (0.8)	0.7 (1.0)	0.4 (0.7)	0.3 (0.7)	0.9 (1.1)	0.3 (0.7)	0.1 (0.4)	**p < 0.001**
Self Harm	0.3 (0.7)	1.8 (1.0)	0.6 (1.1)	0.3 (0.8)	0.9 (1.3)	0.2 (0.7)	0.04 (0.3)	0.9 (1.3)	0.2 (0.7)	0.1 (0.4)	**p < 0.001**
AOD	0.3 (0.8)	0.3 (0.5)	0.6 (0.9)	0.4 (0.8)	0.6 (1.1)	0.1 (0.4)	0.4 (0.9)	0.9 (1.2)	0.4 (0.9)	0.1 (0.4)	**p < 0.001**
Cog	0.3 (0.6)	1.0 (0.8)	0.4 (0.8)	0.4 (0.7)	0.5 (0.8)	0.2 (0.5)	0.3 (0.6)	0.6 (0.8)	0.3 (0.7)	0.3 (0.5)	**0.002**
Phy	0.5 (0.9)	1.3 (1.5)	1.0 (1.2)	0.8 (1.1)	0.8 (1.1)	0.5 (1.0)	0.5 (1.0)	0.8 (1.1)	0.6 (1.0)	0 (0)	**p < 0.001**
Del/Hal	0.1 (0.5)	0.5 (1.0)	0.1 (0.5)	0.1 (0.5)	0.3 (0.8)	0.2 (0.7)	0.04 (0.2)	0.4 (1.0)	0.0 (0.2)	0 (0)	**p < 0.001**
Dep	1.5 (1.0)	2.5 (0.6)	2.4 (1.0)	1.8 (1.0)	2.4 (1.0)	1.5 (1.0)	1.4 (1.1)	2.1 (1.1)	1.4 (1.0)	1.1 (1.0)	**p < 0.001**
Other	2.7 (0.9)	2.5 (0.6)	2.2 (1.1)	2.6 (0.9)	2.0 (1.2)	2.9 (0.9)	2.7 (0.9)	2.5 (1.2)	2.7 (0.9)	3.0 (0.9)	**p < 0.001**
Relat.	1.2 (1.2)	2.5 (1.0)	1.9 (1.1)	1.2 (1.1)	1.5 (1.1)	1.5 (1.2)	0.8 (0.8)	1.8 (1.3)	1.3 (1.2)	0.5 (0.8)	**p < 0.001**
ADL	0.6 (1.0)	1.3 (1.0)	0.8 (1.1)	0.6 (0.9)	0.8 (1.0)	0.8 (1.1)	0.4 (0.8)	1.0 (1.2)	0.5 (0.8)	0 (0)	**p < 0.001**
Living	0.3 (0.8)	1.0 (0.8)	0.5 (0.9)	0.2 (0.6)	0.4 (0.8)	0.3 (0.7)	0.1 (0.4)	0.5 (1.0)	0.2 (0.4)	0 (0)	**p < 0.001**
Occup	0.4 (0.8)	0.5 (0.6)	0.7 (1.0)	0.5 (0.9)	0.7 (1.1)	0.5 (0.9)	0.4 (0.8)	0.7 (1.1)	0.3 (0.7)	0.1 (0.4)	**p < 0.001**

Total	72	4	130	293	1,879	93	47	130	123	8	

Item 1 (Overactive, aggressive, disruptive or agitated behaviour) - mean score for the emotional disorders group are < 1 (except Anxious Avoidant Dependant with four observations). The mean score for the externalising disorders group is > 1.

Item 2 (Non-accidental self-injury) - mean score for the emotional disorders group is < 1 (except Anxious Avoidant Dependant with four observations) and for the externalising disorders group is > 1 with the exception of the ADHD and drug disorders diagnoses.

Item 3 (Problem drinking or drug-taking) -mean scores for the emotional disorders group are < 1 whereas means for externalising disorders is > 0.9, with the exception of the ADHD diagnosis.

Item 6 (Problems associated with hallucinations and delusions) - emotional disorders group was .37 or less, except for Anxious Avoidant Dependent. Externalising disorders are > .34 with the exception of Borderline Personality Disorder which was 0.34.

Item 11 (Problems with living conditions) - the highest mean score in the emotional disorders group (excluding Anxious avoidant dependant) was 0.5 for Post-traumatic stress disorder. This is lower than the mean scores for the externalising disorders group (with the exception of ADHD).

Results indicate that differences exist between the HoNOS item scores for the externalising sub diagnoses. Drug and alcohol disorder patients consistently differ from the other externalising sub-diagnostic groups. Separate analyses show these differences to be significant (p < 0.05 for all but three of the HoNOS items - aggression, relationships and activities of daily living).

For every HoNOS item there are some significant differences between the emotional sub-diagnoses. Notably, specific phobia, social phobia, panic disorder, OCD and agrophobia, have similar profiles with relatively low HoNOS scores (on all items except 'other psychological' and depression). Dysthymia and major depression have similar HoNOS profiles to each other, though worse relationship problems scores are evident in the dysthymic group. The anxious avoidant personality mean scores are relatively higher on a number of scales, including depression, other psychological symptoms, relationships, activities of daily living, self harm and aggression. Patients with a PTSD diagnosis were rated relatively severely on many of the HoNOS items, in particular, depression, 'other psychological', relationships and activities of daily living. They also scored noticeably higher on three of the sub-scales that are more closely aligned with the externalising group (namely aggression, self harm and drug and alcohol).

There are other apparent differences within the externalising group. The outlying HoNOS scores for the diagnosis are ignored due to the small total number (n = 13). Patients diagnosed with borderline personality disorder scored significantly lower on drug and alcohol than the other sub groups. The drug disorder group scored significantly higher on delusions and hallucinations than other constituent sub-diagnoses.

Although the sex ratios differed between the two major groupings (M/F = 1.3 in externalising and 0.6 in emotional groups) analysis by gender shows no significant difference between males and females in HoNOS recorded phenomenology within either the externalising or the emotional group.

## Discussion

Clinicians who identify the DSM diagnoses which get translated into ICD codes for official statistical purposes are likely to have noted the externalising/emotional hypothesis outlined in the introduction. Proponents of this hypothesis suggest that genetic commonalities under-pin two major constructs: externalising and emotional (originally "intrinsic") disorders. They have also, reasonably consistently, related the two categories to three claimed underlying factors or dimensions [12,14,15]. Clinicians are likely to be very reluctant to give up the utilisation of the constituent categories. We believe clinicians support for the proposed 'mega-groupings' would be more forthcoming if its 'face validity' were demonstrated. Symptomatic commonality within those groupings is therefore likely to be an argument for incorporating such a design into the ICD-11 or DSM V. However, the recently released draft of DSM-5 has not used this approach [22].

The present study, which opportunistically utilised routinely collected data, indicates a clear demarcation between the proposed two major groupings on most of the HoNOS recorded items. Of particular note are high mean scores for depression in the emotional disorder grouping, and drug and alcohol consumption, hyperactivity/aggressive behaviour, poor functioning, self harm in the externalising disorder group. However, when examined separately, there are many differences between the emotional disorder constituent diagnoses. This is hardly surprising. If the constituent diagnoses had similar profiles there would be no need for more than one sub category! The number of the individual HoNOS item differences in this study may provoke more focused and thoughtful research into some individual subcategories in an attempt to create sub categories which do have natural boundaries between them and alternative diagnoses. For example, PTSD scores higher than the other emotionality cluster diagnoses particularly on alcohol use and at the higher end on the last three HoNOS scores which all reflect on functionality. This may reflect the classification and validity confusion about PTSD, highlighted by others [9].

The present study represents a compromise between research purity and everyday reality. Clinicians are ultimately pragmatists, more likely to accept classificatory changes which are based on clinical research. They have elsewhere indicated their interest in a degree of rationalising the large number of categories in current classificatory systems [4]. The HoNOS is not an instrument designed specifically for the research of phenomenology or symptoms. However, the analysis utilises a data base of users of a standard public psychiatric service to address issues of bias.

## Conclusions

The analysis of a set of symptom data recorded in everyday, public, psychiatric practice offers some definite, although limited, support to the notion of separate externalising and emotional disorder cluster groupings.

The core, common phenomenology in the externalising disorder group appears to relate to HoNOS items that reflect aggression as well as various measures of functioning. The emotional disorder group appears to be more heterogeneous. Although more difficult to define phenomenologically, depression and relationship difficulties feature strongly.

Further useful work could include examination in credibly large and unselected patient populations of the factor structure demonstrated in non patient samples. Prospective comprehensive trials of the contributions the proposed classification could make to clinical decision making would also help illuminate this area.

## Competing interests

None of the authors has competing interests. Graham Mellsop participates in the meetings of the WHO Advisory Group on the development of ICD-11. The views expressed in this paper are only those of the authors.

## Authors' contributions

The work was conceptualised and initiated by GM; the data management and discussion guided by AB; the statistical analysis by SB. All approved the final paper version.
